# Protracted neuronal maturation in a long-lived, highly social rodent

**DOI:** 10.1371/journal.pone.0273098

**Published:** 2022-09-15

**Authors:** Mariela Faykoo-Martinez, Troy Collins, Diana Peragine, Manahil Malik, Fiza Javed, Matthew Kolisnyk, Justine Ziolkowski, Imaan Jeewa, Arthur H. Cheng, Christopher Lowden, Brittany Mascarenhas, Hai-Ying Mary Cheng, Melissa M. Holmes

**Affiliations:** 1 Department of Cell and Systems Biology, University of Toronto, Toronto, ON, Canada; 2 Department of Psychology, University of Toronto Mississauga, Mississauga, ON, Canada; 3 Department of Biology, University of Toronto Mississauga, Mississauga, ON, Canada; 4 Department of Ecology and Evolutionary Biology, University of Toronto, Toronto, ON, Canada; University of Texas at Austin, UNITED STATES

## Abstract

Naked mole-rats are a long-lived rodent species (current lifespan >37 years) and an increasingly popular biomedical model. Naked mole-rats exhibit neuroplasticity across their long lifespan. Previous studies have begun to investigate their neurogenic patterns. Here, we test the hypothesis that neuronal maturation is extended in this long-lived rodent. We characterize cell proliferation and neuronal maturation in established rodent neurogenic regions over 12 months following seven days of consecutive BrdU injection. Given that naked mole-rats are eusocial (high reproductive skew where only a few socially-dominant individuals reproduce), we also looked at proliferation in brain regions relevant to the social-decision making network. Finally, we measured co-expression of EdU (newly-born cells), DCX (immature neuron marker), and NeuN (mature neuron marker) to assess the timeline of neuronal maturation in adult naked mole-rats. This work reaffirms the subventricular zone as the main source of adult cell proliferation and suggests conservation of the rostral migratory stream in this species. Our profiling of socially-relevant brain regions suggests that future work which manipulates environmental context can unveil how newly-born cells integrate into circuitry and facilitate adult neuroplasticity. We also find naked mole-rat neuronal maturation sits at the intersection of rodents and long-lived, non-rodent species: while neurons can mature by 3 weeks (rodent-like), most neurons mature at 5 months and hippocampal neurogenic levels are low (like long-lived species). These data establish a timeline for future investigations of longevity- and socially-related manipulations of naked mole-rat adult neurogenesis.

## Introduction

Adult neurogenesis is the generation of new neurons in a mature brain followed by subsequent integration into established circuits. In diverse species, the dentate gyrus of the hippocampus is a site of neurogenesis with adult-generated neurons contributing to memory formation for a variety of tasks including spatial memory, fear conditioning, and social recognition, allowing mammals to discriminate changes in the environment and adapt to changing circumstances [1–5]. The subventricular zone also produces adult-generated cells that migrate to the olfactory bulbs [6,7]. Olfactory neurogenesis is related to various social functions including mate selection and recognition of pups and familiar conspecifics [8–11]. Evidence also exists for comparably low level new cell production and survival in a variety of hypothalamic and amygdalar structures related to hunger, social stress, and reproductive functions [12–20]. Thus, adult-generated neurons in various brain regions are important for how animals respond and adapt to their environment throughout life.

Given the importance of adult neurogenesis for organismal plasticity, it is not surprising that patterns, levels, and mechanisms of neuron production are species-specific. Comparative analyses of adult neurogenesis across a variety of mammalian species deepens our understanding of what mechanisms are conserved and therefore how the processes might be similar—or not—in the human brain. For instance, there are similarities in the rates of proliferation and timeline of maturation in larger, longer-lived species that are distinct from what happens in mice and rats. Primate fetuses have a relatively long gestation, which partly accounts for the fact that much of their neurodevelopment and brain mass growth occurs in-utero [21,22]. Allometric studies that compare neurodevelopment by milestones common across species rather than chronological time have found that brains grow at similar rates. However, smaller brains tend to finish growing earlier in development and the rate of brain growth throughout neurodevelopment may be more due to the size of the brain than the size of the animal [23,24]. Comparative analyses found that longer-lived species, such as humans, macaques and sheep, tend to undergo more neurodevelopment by parturition, take longer to reach maturity and produce fewer cells in the adult brain but continue proliferation later in life [21,25–27]. Taken together, the lifespan of a model species is an important variable in making comparative inferences.

The naked mole-rat is a remarkably long-lived rodent species, with the current recorded lifespan in captivity at >37 years [28]. Naked mole-rats’ extended lifespan and slower maturation presents novel opportunities for examining and manipulating neurogenesis at critical periods [29,30]. Further, naked mole-rats are eusocial; they live in colonies of up to 300 individuals consisting of one reproductively active queen, 1–3 breeding males, and all other individuals are reproductively suppressed and socially subordinate [31,32]. Subordinates live their entire lives in a protracted prepubertal state [33–38], presenting the opportunity to understand the interaction between sociality, neuroplasticity and longevity. To date, few papers directly explore neurogenesis in this species. Notably, Orr et al., (2016) [39] identified that newborn naked mole-rats have a larger brain-to-body mass ratio relative to mice, yet it takes 3 months for their brains to reach full mass compared to just 2 weeks in mice]. Work by Penz et al. (2015) [40] identified cell proliferation in the rostral migratory stream is retained even in naked mole-rats as old as ten years of age. Expression of doublecortin (DCX; an endogenous immature neuron marker) is higher and remains more widespread in the piriform cortex of naked mole-rats relative to mice at 1 year of age. While DCX expression is not observed in naked mole-rats by 21 years of age, PSA-NCAM expression (developing and migrating neurons; endogenous plasticity marker) is detectable in the DG at this age [40]. The retention of neoteny is thought to be associated with their longevity [30,41], thus increasing the parallels in naked mole-rat development to humans and other non-human primates. Evidence also suggests that sociality alters DCX and Ki-67 (endogenous proliferation marker) expression in naked mole-rats depending on social context [42–44], and that levels of young neurons are higher relative to the solitary Cape mole-rat [45]. While some properties of naked mole-rat neurogenesis and plasticity have been examined at various developmental stages, a timeline of cell proliferation or neuronal maturation in the naked mole-rat is required to further this branch of research.

Here, we tested the hypothesis that naked mole-rat rates of adult cell proliferation and neuronal maturation are protracted compared to traditional short-lived laboratory rodents. First, we quantified cell proliferation using the thymidine analogue bromodeoxyuridine, an exogenous marker of cell division facilitating birth dating of cells (BrdU; 7 daily injections), over a one year time course in canonical neurogenic regions (dentate gyrus, subventricular zone, olfactory bulb) and potential target sites of migration in subordinate naked mole-rats. We then compared these trajectories to newborn cells after a single 2 hour BrdU injection. Finally, we tested whether these newly born cells developed into neurons by comparing 5-ethynyl-2’deoxyuridine (EdU, a separate thymidine analogue) with DCX and NeuN (neuronal marker) in canonical neurogenic niches.

## Experimental procedures

### Animal care

Naked mole-rats lived in stable colonies (i.e. established breeders were present) housed in large (45.75 cm L × 24 cm W × 15.25 cm H) and small (30 cm L × 18 cm W × 13 cm H) polycarbonate cages connected by plastic tubes (25 cm L × 5 cm D) lined with corncob bedding. Animals were fed sweet potato ad libitum supplemented with wet 19% protein mash (Envigo RMS, Inc.) and kept on a 12:12 light/dark cycle at 28–30 C. All procedures were approved by the University Animal Care Committee and performed in accordance with federal and institutional guidelines.

#### Experiment 1: Cell birth-dating using BrdU

*BrdU injections and tissue collection*. Subordinate animals were injected intraperitoneally (IP) with BrdU (dose = 200mg/kg) [46] once per day for 7 consecutive days. To facilitate comparison with Penz et al. (2015) [40], we used a seven day paradigm of injections. Brain collections occurred at 6 timepoints post-final BrdU injection: 2 hours (N = 12, 5 females, 7 males), 1 week (N = 12, 9 females, 3 males), 3 weeks (N = 11, 6 females, 5 males), 3 months (N = 11, 5 females, 6 males), 5 months (N = 12, 6 females, 6 males), and 1 year (N = 9, 6 females, 3 males). A separate group of animals (N = 12, 5 females, 7 males) was injected once with BrdU and euthanized 2 hours later. Animals used in this study were young non-reproductive adults, aged between 8 months and 2.5 years at injection and aged between 11 months and 3 years at collection. Naked mole-rats reach maturity around 6 months of age and show few differences in neurogenesis up to 3 years of age (the timeframe in which this has been studied) [39]. Further, unlike other rodent models, successful breeding is unpredictable meaning that entire experimental cohorts cannot be generated at one time. To address this potential source of variability, animals of varying ages were yoked across collection times. At its designated collection time point, each animal was euthanized with 2,2,2-tribromoethanol dissolved in 2-methylbutanol (IP; 40mg/100g). Brains were extracted fresh and immersion-fixed for 4 hours in 4% PFA followed by 20% sucrose for 24 hours. Brains were sliced in the coronal orientation on a freezing stage microtome at 30μm into 6 series and stored at -20C in cryoprotectant.

*Immunostaining*. Free-floating sections were pre-treated for antigen-retrieval with sodium citrate buffer (30 minutes at 80 C). Then, sections were incubated for 30 minutes at room temperature with 0.6% H202 to inhibit endogenous peroxidase activity. Next, sections were incubated for 30 minutes at 37°C in 2N HCl for DNA denaturation. Sections were then incubated in a 0.1M borate buffer (pH 8.5) for 10 minutes at room temperature and blocked in 3% normal goat serum in TBS (with 0.1% Triton-X) for 30 minutes at room temperature. Finally, sections were incubated with monoclonal rat anti-BrdU antibody (1:200 in TBS, 48h at 4°C; Accurate Chemicals Cat#OBT0030A) followed by goat anti-rat antibody (1:200 in TBS, 4 h at RT; Vector Laboratories). Cells were visualized for counting with an Avidin-Biotin Complex incubation (ABC; Vector Laboratories) and diaminobenzidine (DAB) reaction. Between all steps, sections were washed for 3 x 5 minutes with 0.1M TBS. Sections were mounted onto slides and dehydrated with an ascending series of ethanol. Slides were cleaned with xylene and cover-slipped using Permount.

*Tissue analyses*. BrdU-expressing cells were counted manually and done blind to the experimental group. Quantification was performed on an Olympus BX51 light microscope with a 40x objective. Counting for each brain region was performed by a single individual, with reliability checks conducted by an additional experimenter. Reported counts for each region are expressed as a density score (the number of cells counted divided by the number of tissue sections analyzed). In canonical regions, 5 sections were counted for the subventricular zone, 3 sections for the olfactory regions (AON, GCL, GloL, OV) and 7 sections for the dentate gyrus (DGhil, DGsgz, DGgcl). In non-canonical regions, 3 sections were counted for the Arc, NAcc, MeA, mPFC, POA and VMH and 5 sections for the BLA, PIC1, PIC2 and PIC3. Analyses were performed bilaterally for the subventricular zone and dentate gyrus. All others were performed unilaterally in order to ensure at least three consecutive sections were sampled per individual.

#### Experiment 2: Phenotyping adult-born cells using EdU, DCX, and NeuN

*EdU injections and tissue collection*. A separate cohort of animals was injected intraperitoneally (IP) with EdU (dose = 25mg/kg) once per day for 7 consecutive days, similar to the dosage used in Penz et al., 2015 [40]. Here, we used EdU instead of BrdU to avoid the denaturation step required for immunostaining, resulting in partial tissue degradation [47], which was interfering with our ability to perform multi-label immunofluorescence. Collections occurred at 5 timepoints post-final EdU injection: 1 week (N = 12, 6 females, 6 males), 3 weeks (N = 12, 6 females, 6 males), 3 months (N = 12, 6 females, 6 males), 5 months (N = 15, 8 females, 7 males), and 12 months (N = 10, 4 females, 6 males). Animals used were young adults whose ages ranged between 1.5 and 5.5 years at injection and between 1.5 and 6 years at collection. While this is relatively double the age of animals used in Experiment 1, these animals are still considered to be relatively young and, as for Experiment 1, age was yoked across collection time. At its designated collection time point, each animal was euthanized with 2,2,2-tribromoethanol dissolved in 2-methylbutanol (IP; 40mg/100g) and transcardially perfused with PBS and 4% paraformaldehyde (PFA). Brains were extracted, post-fixed for 24 hours in 4% PFA followed by 20% sucrose. Brains were sliced on a freezing stage microtome at 30μm into 6 series and stored at -20 C in cryoprotectant.

*Immunostaining*. Tissue sections containing the olfactory bulb, subventricular zone, or dentate gyrus were selected from a single series and processed for triple-label immunofluorescence for EdU, DCX, and NeuN. Sections were anatomically matched across animals and staining was performed on free-floating sections. The sections were washed for 5 minutes in 0.01M PBS and then twice in 3% BSA. Tissue was then permeabilized in 0.5% Triton-X for 20 minutes at RT and washed twice in 3% BSA for 5 minutes. Further steps were performed in a manner to minimize light exposure. Tissue was incubated in a Click-iT reaction cocktail (prepared according to manufacturer’s recommendations; Life Technologies Inc. Cat #C10339) for 30 minutes followed by a 5 minute 3% BSA wash and 0.01M PBS wash. Tissue was blocked in normal horse serum (Vector Laboratories Cat#VECTS2000) in PBS-T (0.1% Triton-X, 1X PBS, 10% NHS) for 1 hour at room temperature. We used NHS rather than normal donkey serum because pilot work showed better signal to noise ratio in our tissue. Tissue was incubated in primary antibody (monoclonal mouse anti-NeuN 1:1000, EMD Millipore Cat#MAB377; polyclonal rabbit anti-DCX 1:250, Abcam Cat#AB18723) for 24 hours at 4°C. Following a 5 minute wash in PBS-T, tissue was incubated for 2 hours at RT in secondary antibody in NHS/PBS-T (donkey anti-mouse 488 1:1000, Life Technologies Cat#A21206; donkey anti-rabbit 647 1:500; EMD Millipore Cat#AP182SA6). Tissue was mounted onto slides and cover-slipped with Dako fluorescent mounting medium (Agilent Cat#S2023).

*Tissue analyses*. For each region of interest, every visible EdU+ cell was phenotyped and categorized as either EdU+, EdU/DCX+, EdU/NeuN+ or EdU/DCX/NeuN+. All phenotyping was done manually and by an experimenter blind to the experimental group. Analysis was performed on Zeiss LSM 700 and Zeiss LSM 800 confocal microscopes at 40x magnification. Confocal images were captured in separate channels per fluorophore. Settings were kept consistent for each run of staining including gain, pinhole, and filters. Z-stacks were set to 2μm. Images were analyzed on the ImageJ program. Counts for each cell type are reported as a density score (number of cells counted divided by number of tissue sections analyzed). Two sections were used for the subventricular zone, 3 sections for the olfactory bulb, and 4 sections for the dentate gyrus. All analyses were done bilaterally.

### Statistical analyses

All statistical analyses were performed in R [48]. Graphics were produced using ggplot2 [49]. Linear mixed-effects models were run using nlme. Linear mixed-effects models effectively deal with unbalanced group numbers and allowed us to account for colony variation within and between groups. Multiple-testing correction of models was performed using the p.adjust() function in the base R package using the argument for false discovery rate. Post-hocs were performed using Tukey’s method in emmeans to account for multiple testing correction [50].

### Experiment 1

Models were assessed for collinearity amongst predictor variables prior to fitting using the vif() function in the usdm package (VIF cut-off < 4) [51]. Models were fit for collinearity between the predictor variables collection time, sex and brain region; sex was then collapsed (see Results for statistics).

For each brain region, a linear mixed-effects model was fit with density (number of cells counted divided by the number of counted sections) as the response variable, collection time as the predictor variable, and colony as the random error variable. Residuals were tested for normality, resulting in log transformation of density for the following models: Arc, AON, DGhil, DGgcl, DGsgz, GCL, GloL, meA, mPFC, OV and VMH. Multiple-testing correction was performed using false discovery rate on the main effect of collection time for all brain regions (canonical and non-canonical). Within each brain region, Tukey post-hoc adjustments were used.

To infer whether cells were born in a brain region or rapidly migrating, we performed Welch two-sample t-tests between BrdU density in a brain region 2 hours after a single injection and 2 hours after the last of 7 daily injections. Multiple-testing correction was performed on all brain regions (canonical and non-canonical) for collection time.

### Experiment 2

We first tested whether data for markers BrdU and EdU for Experiments 1 and 2, respectively, were consistent. For Experiment 2, EdU counts were totaled for EdU+, EdU/DCX+ and EdU/NeuN+ counts. No EdU/DCX/NeuN+ cells were identified. Datasets were independently scaled using z-scores. The normalized density counts between datasets were compared using a linear mixed-effects model where collection time and marker were response variables, co-varied by brain region. No significant difference was observed for the main effect of markers in this model (statistics reported in Results).

Each canonical brain region (subventricular zone, AON, OV, GloL, DGhil, DGsgz and DGgcl) was tested using a linear mixed-effects models with density counts as the response variable, collection time as the predictor variable and colony as the random error variable. Residuals were tested for normality. EdU+, EdU/DCX+ and EdU/NeuN+ counts were analyzed using separate models. Multiple-testing correction was performed using false discovery rate on the main effect of collection time for all brain regions within a marker type, Tukey post-hoc adjustments were used. Proportions of EdU+, EdU/DCX+ and EdU/NeuN+ density were calculated relative to the total EdU+ density per individual. These proportions were averaged across individuals within a collection time.

Due to very low cell numbers in non-canonical regions, we were unable to phenotype cells in a quantitative manner. Instead, we took a qualitative approach to determine whether we could identify any EdU/DCX+, EdU/NeuN+ or Edu/DCX/NeuN+ cells in these regions. We looked at sections at the 5 and 12 month collection times, as these are the times at which we identified EdU/DCX+ and EdU/NeuN+ cells in the canonical brain regions. While we identified EdU+ cells, we did not find evidence that these cells co-expressed DCX or NeuN.

## Results

### Experiment 1

#### A complete summary of all statistics for both experiments can be found in the Supplementary Files

Model fitting found no collinearity between the predictor variables of interest: collection time (VIF = 1.02), sex (VIF = 1.00), and brain region (VIF = 1.01). As in Faykoo-Martinez et al., 2018 [51], we collapsed sex as it was not a significant predictor variable (brain region: F(17,1025) = 46.4, p<0.001; collection time: F(5,1025) = 10.4, p<0.001; sex: F(1,1025) = 0.07, p = 0.79; collection time by sex interaction: F(5,49) = 0.65, p = 0.660).

**Fig 1:**
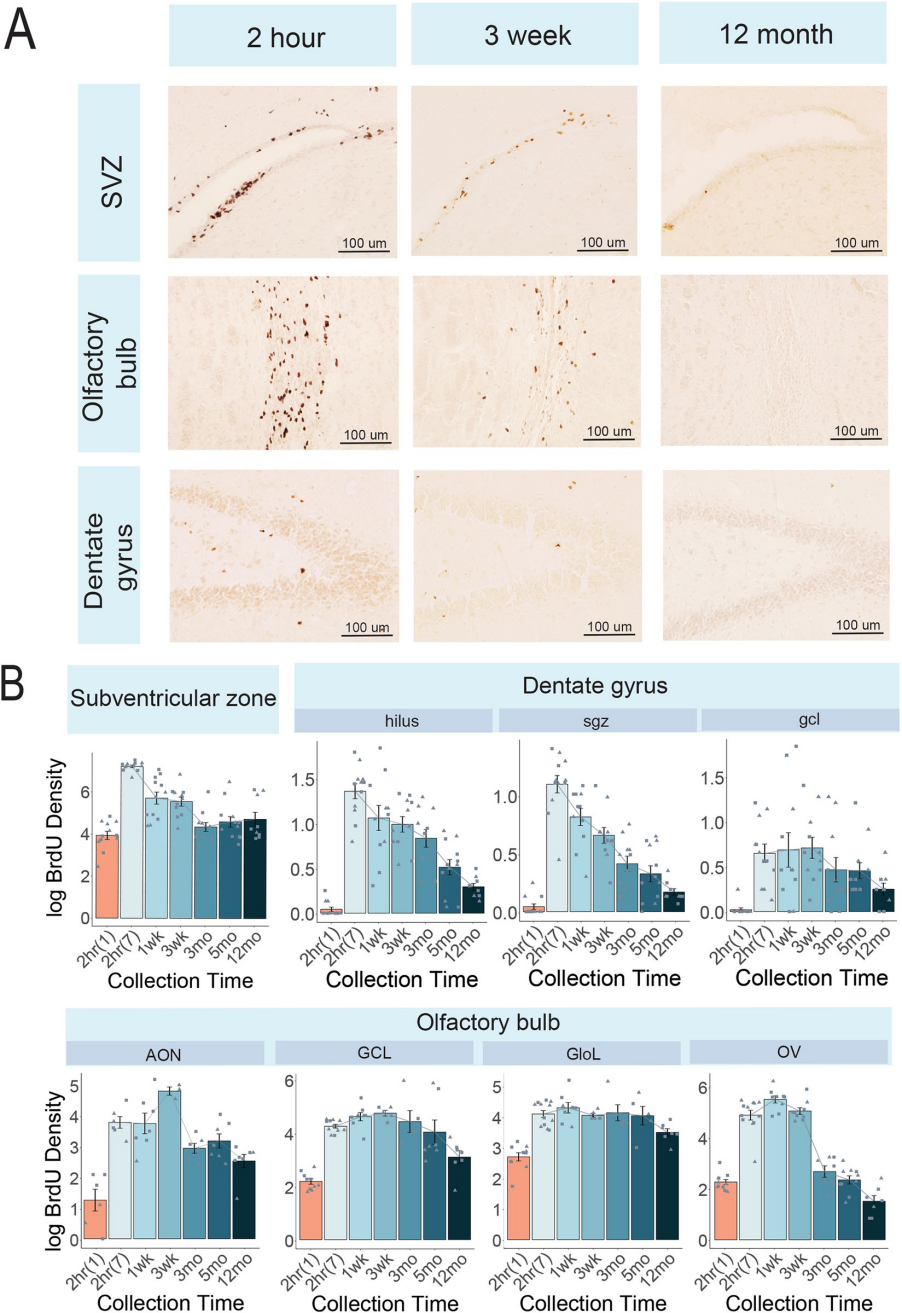
Cell proliferation in canonical neurogenic niches. A) Photomicrographs of the subventricular zone (SVZ), olfactory bulb, and dentate gyrus at representative timepoints after the last of 7 BrdU injections: 2 hour, 3 week, and 12 month. B) Bar plots of density (+/- SEM) across timepoints in the subventricular zone and subregions of the olfactory bulb (anterior olfactory nucleus, AON; granule cell layer, GCL; glomerular layer, GloL; and olfactory ventricle, OV) and the dentate gyrus (hilus; sub-granular zone, sgz; granular cell layer, gcl). 2hr(1) = animals collected after a single injection and 2hr(7) = animals collected after 7 injections. Density was averaged over the following number of sections per region: Subventricular zone = 5 sections, olfactory bulb = 3 sections, dentate gyrus = 7 sections. Circle = male; triangle = female.

*Canonical regions (Fig 1).* A significant effect of collection time in the subventricular zone (F(5,52) = 45.3, padj<0.001) revealed BrdU cell density was highest at 2 hours following the last BrdU injection relative to all other timepoints (1 week, p<0.001; 3 weeks, p<0.001; 3 months, p<0.001; 5 months, p<0.001; 12 months, p<0.001). BrdU cell density did not change between 1 and 3 weeks (p = 0.803), with the next decrease in cell density occurring after 3 months (p = 0.016) and 5 months (p = 0.047) relative to 1 week.

BrdU density counts in the AON (F(5,22) = 8.01, padj<0.001) decrease overall from 2 hours to 12 months (p = 0.026), 1 week to 12 months (p = 0.004) and between 3 weeks and 3 months (p = 0.006), 5 months (p = 0.009) and 12 months (p<0.001). A similar pattern was observed in the GCL (F(5,28) = 5.49, padj = 0.001) with a decrease between 2 hours to 12 months (p = 0.022), 1 week to 12 months (p = 0.001), 3 weeks to 12 months (p = 0.003) and 3 months to 12 months (p = 0.015). In the GloL (F(5,27) = 3.68, padj = 0.032), the only significant pairwise comparison was between 1 week and 12 months (p = 0.004). After 7 days of injections, no change in BrdU density was observed in the OV (F(5,39) = 84.2, padj<0.001) between 2 hours and other collection times until 3 months (p = 0.007); density remained low at 5 months (p<0.001) and 12 months (p<0.001). BrdU density in the OV further decreased between 3 weeks and 5 months (p<0.001) and 12 months (p<0.001), between 3 months and 12 months (p = 0.003) and finally, between 5 months and 12 months (p = 0.028).

A significant effect of collection time in the DGsgz (F(5,51) = 23.0, padj<0.0001) revealed BrdU density peaked at 2 hours relative to all other collection times (1 week, p = 0.056; 3 weeks, p<0.001; 3 month, p<0.001; 5 months, p<0.001; 12 months, p<0.001). A statistically significant decline in density was observed between 1 week and 3 months (p = 0.002), but not 1 week and 3 weeks (p = 0.511). A similar pattern of collection time in the DGhil (F(5,51) = 14.9, padj<0.001) revealed a decrease in density between 2 hours and 3 months (p = 0.004), 5 months (p<0.001) and 12 months (p<0.001), between 1 week and 5 months (p = 0.001) and 12 months (p<0.001), between 3 weeks and 5 months (p = 0.007) and 12 months (p<0.001), between 3 months and 12 months (p = 0.005) and no difference in BrdU density between 5 months and 12 months (p = 0.569). No significant effect of collection time on BrdU density was observed in the DGgcl (F(5,51) = 2.04, padj = 0.09).

**Fig 2:**
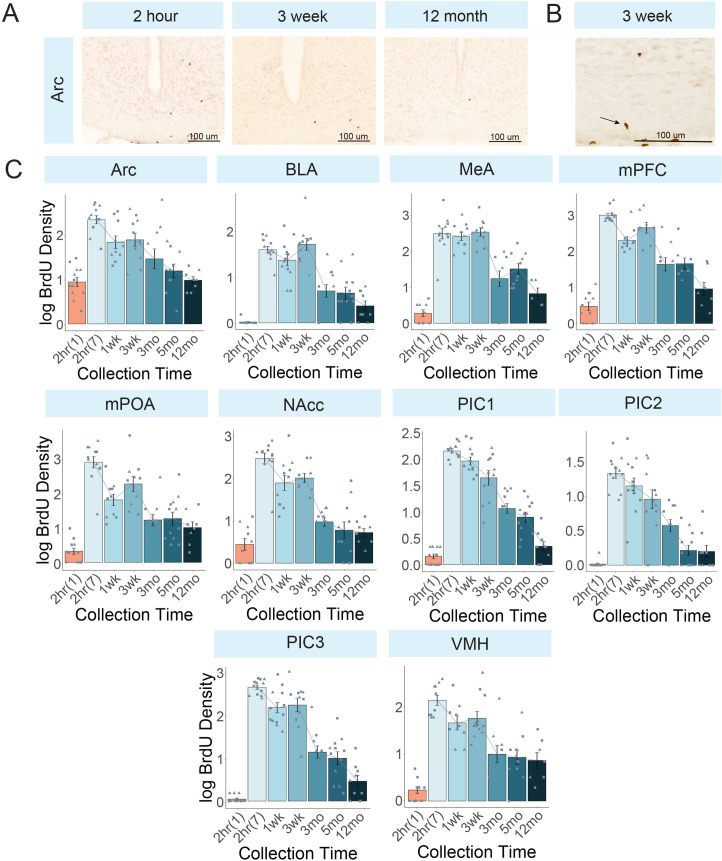
Cell proliferation in non-canonical neurogenic niches. A) Photomicrographs of a representative brain region, the arcuate nucleus of hypothalamus, at representative timepoints after the last of 7 BrdU injections: 2 hour, 3 week, and 12 month. B) Higher magnification image (400x) of BrdU cells in the arcuate nucleus of the hypothalamus at 3 weeks. C) Bar plots of density (+/- SEM) across timepoints in non-canonical neurogenic regions. 2hr(1) = animals collected after a single injection and 2hr(7) = animals collected after 7 injections. The number of sections averaged to calculate density are in parentheses following the abbreviation: Arc = arcuate nucleus of the hypothalamus (3 sections); BLA = basolateral amygdala (5 sections), MeA = medial amygdala (3 sections), mPOA = medial pre-optic area (3 sections), NAcc = nucleus accumbens (3 sections), PIC1 = piriform cortex–layer 1 (5 sections), PIC2 = piriform cortex–layer 2 (5 sections), PIC3 = piriform cortex–layer 3 (5 sections), VMH = ventromedial hypothalamus (3 sections). Circle = male; triangle = female.

*Non-canonical regions (Fig 2).* BrdU density counts in the non-canonical regions were lower than those observed in the canonical regions, however patterns across timepoints remained similar.

A significant effect of collection time in the Arc (F(5,46) = 11.4, padj<0.001) revealed a relatively steady decline across timepoints. Statistically, a decrease in BrdU density was observed between 2 hours and 3 months (p<0.001), 5 months (p<0.001) and 12 months (p<0.001). There was no decrease between 1 week and 3 weeks (p = 0.999), or 3 months (p = 0.496), but it did decrease by 5 months (p = 0.041) and 12 months (p = 0.005). Another decrease was observed between 3 weeks and 5 months (p = 0.017) and 12 months (p = 0.002).

A similar pattern was observed in the BLA (F(5,52) = 13.8, padj<0.001) were a decrease in BrdU density was observed between 2 hours and 3 months (p = 0.004), 5 months (p = 0.003) and 12 months (p<0.001). Similarly, while no change in BrdU density was observed between 1 week and 3 weeks (p = 0.075) or 3 months (p = 0.052), a statistically significant drop was observed by 5 months (p = 0.041) and 12 months (p = 0.002). Further, another decrease occurred between 3 weeks and 3 months (p<0.001), 5 months (p<0.001) and 12 months (p<0.001). This pattern was also observed in the MeA (F(5,428) = 20.218.1, padj<0.0001)), with a drop in density between 2 hours and 3 months (p<0.001), 5 months (p<0.001) and 12 months (p<0.001), between 1 week and 3 months (p<0.001), 5 months (p<0.001) and 12 months (p<0.001) and between 3 weeks and 3 months (p<0.001), 5 months (p<0.001), 12 months (p<0.001).

The mPFC (F(5,41) = 27.7, padj<0.0001) observed a decrease between 2 hours and 1 week (p = 0.010), but not 3 weeks (p = 0.547), before decreasing again at 3 weeks (p<0.001), 3 months (p<0.001), 5 months (p<0.001) and 12 months (p<0.001). No change was observed between 1 week and 3 weeks (p = 0.507), however density decreased by 3 months (p = 0.010), 5 months (p = 0.026) and 12 months (p<0.001). Further, decreases in density were observed between 3 weeks and 3 months (p<0.001), 5 months (p<0.001) and 12 months (p<0.001), between 3 months and 12 months (p = 0.020) and between 5 months and 12 months (p = 0.023). Like the mPFC, the NAcc (F(5,462) = 20.8, padj<0.001) peaked at 2 hours (1 week, p = 0.004; 3 weeks, p = 0.009; 3 months, p<0.001; 5 months, p<0.001; 12 months, p<0.001). No difference between 1 week and 3 weeks (p = 1.000) was observed but density decreased after 3 months (p = 0.005), 3 months (p = 0.003), 5 months (p = 0.003) and 12 months (p = 0.002). Density at 3 weeks decreased after 3 months (p = 0.008), 5 months (p = 0.005) and 12 months (p = 0.004); no significant changes were seen between 3 and 12 months.

Density in the PIC1 (F(5,52) = 37.8, padj<0.001) did not decrease after 1 week (p = 0.371), but decreased after all following timepoints (3 weeks, p<0.001; 3 months, p<0.001; 5 months, p<0.001; 12 months, p<0.001). Similarly, density decreased between 1 week and 3 months (p<0.001), 5 months (p<0.001) and 12 months (p<0.001), between 3 weeks and 3 months (p = 0.002) and 5 months (p<0.001), but plateaued after 3 months. BrdU density in the PIC2 (F(5,52) = 14.5, padj<0.001) remained consistent between 2 hours and 1 week (p = 0.961) and 3 weeks (p = 0.316) before gradually declining (non-significant change) after 3 months (p<0.001), 5 months (p<0.001) and 12 months. Density decreased between 3 weeks and 5 months (p<0.001), but not between 3 months to 12 months. In the PIC3 (F(5,5249) = 29.7, padj<0.001), density peaked at 2 hours relative to all other groups (1 week, p = 0.004; 3 weeks, p = 0.042; 3 months, p<0.001; 5 months, p<0.001; 12 months, p<0.001). No difference was observed between 1 week and 3 weeks (p = 0.971) but density decreased after 3 months (p<0.001) and remained unchanged thereafter.

BrdU density in the mPOA (F(5,48) = 14.0, padj<0.001) peaked at 2 hours, declined by 1 week (p<0.001), with no difference between 1 week and 3 weeks (p = 0.366) before declining again by 3 months relative to 3 weeks (p = 0.029), with no changes over the next year. For BrdU density in the VMH (F(5,42) = 12.5, padj<0.001), no changes were observed across the first 3 weeks, however density decreased by 3 months (p<0.001), 5 months (p<0.001) and 12 months (p<0.001). Density decreased between 3 weeks and 3 months (p = 0.004) before remaining unchanged to 12 months.

*2 hour post-single BrdU injection*. Two hours following a single injection of BrdU, the average density count was 59.00(+/-9.81) in the subventricular zone. Average density counts in the olfactory bulb were 3.555 (+/-1.27) in the AON, 8.300(+/-0.99) in the GCL, 14.83(+/-1.73) in the GloL and 9.272 (+/-1.22) in the OV. Counts were substantially lower in the dentate gyrus at 0.048(+/-0.03) in the DGhil, 0.048(+/-0.03) in the DGsgz and 0.012(+/-0.01) in the DGgcl. Density counts in the noncanonical regions varied as follows: Arc, 1.694 (+/-0.27); BLA, 0.017 (+/-0.02); MeA, 0.370(+/-0.13); mPFC, 0.727(+/-0.20); mPOA, 0.444(+/-0.14); NAcc, 0.700(+/-0.24); PIC1, 0.183(+/-0.05); PIC2, 0.017(+/-0.02); PIC3, 0.050(+/-0.03); VMH, 0.300 (+/-0.10). In each region, there were significantly more labelled cells following 7 injections compared to 1 injection. All adjusted p-values were less than <0.05; a full summary of results can be found in the Supplementary Files.

### Experiment 2

#### A complete summary of all statistics can be found in the Supplementary Files

A comparison of the BrdU and EdU data revealed a main effect of collection time (F(1,338) = 5.43, p = 0.020) but not marker (F(1,7) = 0.000, p = 1.000) or interaction of collection time and marker (F(1,338) = 26.8, p = 0.115), suggesting the BrdU and EdU datasets are capturing the same effects of collection time.

**Fig 3:**
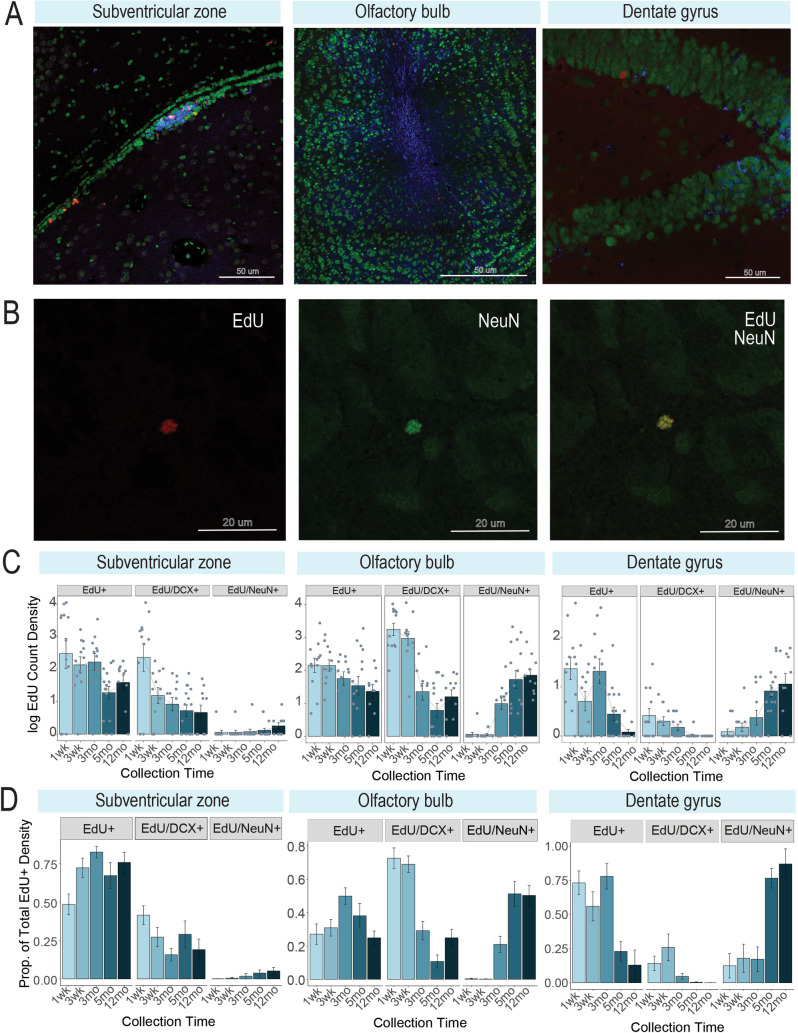
Cell maturation in canonical neurogenic niches. A) Representative triple label immunofluorescent photomicrographs of the subventricular zone (animal collected after 1 week), olfactory bulb (animal collected after 5 months) and dentate gyrus (animal collected after 5 months) (left to right). EdU = red, NeuN = green, DCX = blue. B) Representative photomicrographs of a cell co-expressing EdU (red) and NeuN (green). C) Bar plots of density (+/- SEM) for EdU+ only, EdU/DCX+, and EdU/NeuN+ density in the subventricular zone, olfactory bulb and dentate gyrus (left to right). Tissue is from the OB of an animal in the 5 month collection group. D) Proportions of density (+/- SEM) for EdU+, EdU/DCX+, and EdU/NeuN+ cells (out of total EdU+ density, calculated within an individual) per brain region. Density is averaged over the following number of sections per region: Subventricular zone = 2 sections, olfactory bulb = 3 sections, dentate gyrus = 4 sections.

EdU+ cell density in the subventricular zone (F(4, 54) = 7.20, padj = 0.003; Fig 3A) peaked at 1 week and subsequently plateaued (3 weeks, p = 0.016; 3 months, p = 0.037; 5 months, p<0.001; 12 months, p<0.001). The same pattern was observed for EdU/DCX+ cell density (F(4, 54) = 11.9, padj<0.001) in this region where cells peaked at 1 week (relative to 1 week: 3 weeks, p<0.001; 3 months, p<0.001; 5 months, p<0.001; 12 months, p<0.001. No effect of collection time was found for EdU/NeuN+ cells (F(4,54) = 0.93, p = 0.452), yet low numbers of 1–3 cells were observed in several individuals per collection time starting as early as 1 week. No EdU/DCX/NeuN+ cells were observed.

EdU+ cell density in the olfactory bulb did not vary across collection time (F(4, 54) = 1.89, padj = 0.125; Fig 3B), however a significant effect of collection time on EdU/DCX+ (F(4, 54) = 20.0, padj<0.001) and EdU/NeuN+ cells (F(4, 54) = 6.78, padj<0.001) was detected. Edu/DCX+ cells peaked at 1 week relative to all other timepoints (3 weeks, p = 0.044; 3 months, p<0.001; 5 months, p<0.001; 12 months, p<0.001). EdU/DCX+ cell density was also higher at 3 weeks relative to other timepoints (3 months, p<0.001; 5 months, p<0.001;12 months, p<0.001), with counts plateauing after 3 months. EdU/NeuN+ cells appeared as early as 3 weeks, however the bulk of these cells appeared at 5 months (relative to 3 weeks: 5 months, p = 0.001; 12 months, p = 0.042). No EdU/DCX/NeuN+ cells were observed.

A significant effect of collection time in the dentate gyrus was observed for EdU+ cells (F(4, 54) = 6.25, padj<0.001; Fig 3C), EdU/DCX+ cells (F(4, 54) = 4.09, padj = 0.006) and EdU/NeuN+ cells (F(4,54) = 8.89, padj<0.001). EdU+ cells peaked first at 1 week (relative to 5 months, p = 0.010, and 12 months, p = 0.005), with a second peak at 3 months (relative to 5 months, p = 0.012, and 12 months, p = 0.006). EdU/DCX+ cells peaked at 1 week, with a significant drop observed by 5 months (p = 0.008) and 12 months (p = 0.016). EdU/NeuN+ cells appeared in the dentate gyrus as early as 1 week in some animals, with the majority maturing by 5 months (p = 0.004) or 12 months (p = 0.001); another increase occured between 3 months and 12 months (p = 0.006). No significant loss occurred between 5 and 12 months (p = 0.597). No EdU/DCX/NeuN+ cells were observed.

Proportions of EdU/DCX+ cells in the subventricular zone peak at 1 week and 3 weeks, making a sharp decline by 3 months and remain low thereafter. Alternatively, EdU/NeuN+ proportions emerge at 3 months. In the olfactory bulb, large proportions of EdU+ cells remain solely EdU+ across timepoints measured; Edu/DCX+ cells also remain relatively consistent across timepoints. EdU/NeuN+ cells increased across timepoints but were few. In the dentate gyrus, EdU+ cells plummeted at 5 months and remained low. Similar to the olfactory bulb, EdU/DCX+ cell proportions dropped by 3 months and by 5 months, EdU/NeuN+ proportions increased and remained elevated at 12 months. While few adult-generated cells expressed EdU and NeuN in the olfactory bulb, >50% of EdU+ cells in the subventricular zone and dentate gyrus expressed NeuN+ at 5 and 12 months, implying their maturation.

## Discussion

Here, we tracked the birth of newly-born cells in the brains of adult naked mole-rats across a 12 month period. We looked beyond the hippocampus, subventricular zone, and olfactory bulb into regions of the brain that are not typical neurogenic regions but contribute to the remarkable social adaptations of this species. We then determined how long it takes for these newly proliferating cells to develop into neurons. Consistent with previous work in this species, levels of adult cell proliferation were low in the DG, yet were high in the subventricular zone [40,45]. Cell proliferation was high in the subventricular zone only 2 hours after a single injection indicating cells were born here. Most of these newly-born cells migrated to the olfactory bulb, presumably through the rostral migratory stream. Hippocampal adult neurogenesis levels (born in the DGsgz and migrating inwards to the DGhil or outwards to the DGgcl) were low. Cell proliferation was highest in regions after the first timepoint post-BrdU or -EdU injection, with labeled cells gradually declining, but surviving, until at least 12 months later. Newly-born cells in the olfactory bulb and dentate gyrus matured into neurons by 3–5 months and persisted for at least 12 months. This is consistent with findings that naked mole-rats can preserve stable neurogenic potential far longer than mice [40]. In non-canonical regions of neurogenesis we observed low levels of newly-born cells. However, we did not find evidence that these cells matured into neurons.

By tracking cell proliferation and maturation in the adult naked mole-rat, we further demonstrate the naked mole-rat follows a developmental trajectory that is more similar to other long-lived species than it is to traditional laboratory rodents [39]. DCX expression in the naked mole-rat differed between regions, but peaked across all regions at 1 week (Fig 3). DCX expression decreased after 1 week in the subventricular zone, decreased after 1 and 3 weeks in the olfactory bulb, and in dentate gyrus a significant decline was observed at 5 months. A cross-species analysis of DCX+ cells in the dentate gyrus [25] found neurogenesis is strongly correlated to lifespan, but not other biological factors (e.g. body mass, metabolism). The naked mole-rat timeline of olfactory bulb and dentate gyrus neurogenesis resembled that of larger, long-lived animals such as sheep or macaques or Chiroptera species (e.g. brown bat) [26], but not similarly-sized rodents (e.g. mice, rats) [52–55]. In mice and rats, neurons born in the olfactory bulb take approximately 1 month to reach maturity as compared to 3 months in sheep and macaques [52–56]. However, some new neurons appeared as early as 3 weeks in the olfactory bulb of naked mole-rats, suggesting there may be some overlap with what is seen in other rodents. Hippocampal DCX expression in mice/rats declines sharply between 2–3 weeks and mostly disappears by 1 month [53,57]. In contrast, macaques express DCX at 2 weeks, peak around 1.5 months and plateau by 6 months [52–56]. Sheep have a low but steady EdU/DCX co-expression from 1 month to 8 months [52–56]. This long-term stability of naked mole-rat newly-born cells from subventricular zone is posited to be related to protection against DNA damage, which is associated with its longevity [58]. While neuronal maturation timelines in the naked mole-rat appear to be a blend of what is known about long-lived and rodent species, migration patterns of newly-born cells appear consistent with other Rodentia species.

The large majority of newly-born cells appeared to follow the rostral migratory stream characterized in other rodents and is consistent with a previous report in naked mole-rats [39]. Newly born cells were highest in the subventricular zone 2 hours following the last injection (Fig 1C). This number dropped drastically following 1 week post-final injection and remained low across the 12 month period we tracked. These levels declined between 3 weeks and 3 months in OV. It appears these cells then migrated into the AON by 3 weeks and remained there (Fig 1C). Two hours following a single injection, the subventricular zone retained the highest number of newly-born cells compared to olfactory bulb regions, yet quantifiable BrdU expression in the olfactory bulb implies some of these cells were also born there. Consistent with previous reports using endogenous markers in naked mole-rats, very low levels of BrdU-labeled cells were observed in the dentate gyrus (Fig 1C). Adult hippocampal neurogenesis has previously been identified as occurring at low levels in African mole-rats, including naked mole-rats, despite their sociality [30,45]. Given that we did not find a change in BrdU-expressing cells in the DGgcl across timepoints, it is possible cells migrated into the DGhil but not the DGgcl in an unmanipulated subordinate naked mole-rat. These findings demonstrate adult hippocampus neurogenesis is more consistent with the long-lived brown bat species, which show minimal [or no] adult hippocampal neurogenesis [58], than they are with other rodent species.

Identifying where these newly-born cells migrate to and how they mature is an important component of understanding adult-born cells. This can be achieved through experiments exploring the functional importance of neurogenesis in the naked mole-rat. Our previous work on the interaction of social dynamics and neurogenesis has revealed that the sex of a housing partner alters novel cell birth [43]. Opposite-sex pair housing, relative to same-sex pair housing, is associated with a decrease in cortisol and aggression, increased huddling, and increased Ki-67/DCX expression in the DGgcl [43]. In the current experiment, we saw differences between cell proliferation across timepoints despite not having a social manipulation. Housing with a female (regardless of sex) results in higher DCX expression in the BLA compared to colony-housed subordinates. This may be associated with increased risk assesment [43] and the findings demonstrate social stressors do not always downregulate neurogenesis. Risk assessment within the colony structure is important to avoid aggressive encounters. The relationship between risk assessment and adult neurogenesis is further substantiated by increased DCX expression in the BLA, dentate gyrus, and PIC of subordinates relative to socially-dominant breeders [42]. Given that brain regions do not work in isolation, it is important to consider brain regions involved in circuitry relevant to social decision-making [59].

In other species, there is evidence for new cell production in non-traditional adult neurogenic niches, such as those involved in the social decision-making network (e.g.: hippocampus, amygdalar structures, nucleus accumbens, hypothalamus, etc.) [12–19,60]. We previously demonstrated differences in coordinated neural activity in hippocampal and olfactory structures when subordinates are presented with familiar or unfamiliar animals (using the early immediate marker c-Fos), although activity in these regions is distinct from the social decision-making network [61]. The functional purpose of newly-born cells in non-traditional niches is not as well understood as those found in the olfactory bulb/dentate gyrus. However, there is evidence that rates of proliferation and survival are affected by socio-environmental factors [13,16,18,62–66]. Voles are a notable example. Meadow voles and prairie voles (who are polygamous and monogamous, respectively) show species-specific responses of cell proliferation, survival and neuronal maturation in response to sociosexual manipulations [13,16,65]. For example, sociosexual interaction alters cell proliferation and birth of neurons in hypothalamic and amygdalar structures in the monogamous prairie vole [13]. Our 2 hour single injection collection time indicates that there are low levels of newly-born cells in these regions and that newly-born cells persist into adulthood. This presents an intriguing research question of how neurogenesis in these non-canonical niches might shape behavioral responses to the shifting social environment of the colony in naked mole-rats.

Here, we provide evidence that cells born in adult naked mole-rats matured into neurons by 3–5 months. These maturation patterns reflect long-lived species more-so than other rodent species (e.g., mice/rats). We acknowledge that we are singularly profiling subordinates–these individuals are pre-pubertal, pre-reproduction and (for the most part) are in early adulthood. Several key questions require further exploration in the field of naked mole-rat adult cell proliferation. First, what proportion of these newly-born cells mature into non-neuronal cell-types (e.g., astrocytes, oligodendrocytes, etc)? This work would be complemented by investigations into the plasticity of newly-born cells in the context of opportunities where transition within the social hierarchy is possible. Next, do these cells integrate into social decision-making circuits in response to the environment (e.g. when a change in social rank is possible)? Finally, in order to discern how adult-generated cells contribute to behavioral/physiological development, it will be important to develop a species-specific catalog of neurogenic markers. This will become possible as species-specific single-cell atlases continue to emerge in the literature and can help us to identify which of the established markers of proliferating/developing cells exist in the naked mole-rat. These data will contribute to our understanding of longevity and sociality in mammalian species and how this biomedical model can contribute to our understanding of plasticity and aging in the brain.

## Supporting information

S1 File (XLSX)

S1 Raw data (XLSX)

## Abbreviations

AON: anterior olfactory nucleus
Arc: arcuate nucleus of the hypothalamus
BLA: basolateral amygdala
DGhil: hilus of the dentate gyrus
DGgcl: granule cell layer of the dentate gyrus
DGsgz: subgranular zone of the dentate gyrus
GCL: granule cell layer of the olfactory bulb
GloL: glomerular layer of the olfactory bulb
MeA: medial amygdala
mPFC: medial pre-frontal cortex
mPOA: medial pre-optic area
NAcc: nucleus accumbens
OV: olfactory ventricle
PIC1: piriform cortex – layer 1
PIC2: piriform cortex – layer 2
PIC3: piriform cortex – layer 3
PVN: paraventricular nucleus of the hypothalamus
VMH: ventromedial hypothalamus
